# Laboratory scale evaluation of the feasibility of locally found bladderworts as biological agents to control dengue vector, *Aedes aegypti* in Sri Lanka

**DOI:** 10.1186/s12870-023-04454-x

**Published:** 2023-10-04

**Authors:** Nayana Gunathilaka, Ravina Perera, Deepika Amerasinghe, Lahiru Udayanga

**Affiliations:** 1 Department of Parasitology, Faculty of Medicine, University of Kelaniya, Ragama, Sri Lanka; 2 Department of Zoology and Environmental Management, Faculty of Science, University of Kelaniya, Dalugama, Sri Lanka; 3 Department of Biosystems Engineering, Faculty of Agriculture and Plantation Management, Wayamba University of Sri Lanka, Makadura, Sri Lanka

**Keywords:** Aquatic carnivorous plant, *Utricularia*, Predation, Mosquito larvae, Control

## Abstract

**Background:**

The carnivorous genus *Utricularia* also includes aquatic species that have the potential to trap a wide range of prey, leading its death due to anoxia. However, the effectiveness of such an approach with carnivorous plants for vector control has not been evaluated in Sri Lanka.

**Methods:**

Early instar (i & ii) and late instar (iii & iv) larvae of *Aedes aegypti* were exposed to locally found bladderwort (*U. aurea* Lour and *Utricularia* sp.). The experimental design was set with 10 larvae (both early and late instars separately) in 250 mL of water with bladderworts containing approximately 100 bladders in plant segments of both species, separately. Each treatment and control were repeated 50 times. The survival status of larvae was recorded daily until death or adult emergence. The larvae found whole or partially inside the bladders were attributed to direct predation. The Cox-regression model and Mantel-Cox log rank test were carried out to assess the survival probabilities of larvae in the presence of two bladderworts separately.

**Results:**

The highest predation was observed when using early instar larvae in both *U. aurea* (97.8%) and *Utricularia* sp. (83.8%). The mortality caused due to predation by *U. aurea* was observed to be significantly higher according to the Mantel-Cox log-rank test (HR = 60.71, CI; 5.69–999.25, *P* = 0.004). The mortality rates of late instar stages of *Ae. aegypti* were observed to be lower in both *U. aurea* (82.6%) and *Utricularia* sp. (74.8%). Overall, the highest predation efficacy was detected from *U. aurea* (HR = 45.02; CI: 5.96–850.51, *P* = 0.017) even in late instar stages. The results suggested the cumulative predation in both plants on *Ae. aegypti* larvae was > 72%.

**Conclusions:**

*Utricularia aurea* is a competent predator of *Ae. aegypti* larvae. Further, it is recommended to evaluate the feasibility of this plant to be used in the field as a control intervention in integrated vector management programmes.

**Supplementary Information:**

The online version contains supplementary material available at 10.1186/s12870-023-04454-x.

## Background

Dengue has become a formidable challenge of public health in developing countries with rapid urbanization and infrastructure despite control efforts and continuous management programmes [1]. The lack of an effective vaccine or drug for this disease is the major hurdle in the control of dengue disease [2]. Hence, vector control has received wider attention in control programmes to suppress the vector population thereby disrupting disease transmission [3, 4].

As a result of the adverse effect allied with chemical-based vector control methods in terms of developing resistance to insecticides by vectors and detrimental impacts to the environment and other non-targeting organisms, many research studies have focused on evaluating environmentally friendly vector control approaches in recent years [5, 6]. The use of suitable alternative forms of natural enemies as biological forms of control has been adjoined to integrated vector management. Biological approaches such as larvivorous fish, copepods, dragonfly nymphs, and endoparasitic ciliates have been tested and applied in Sri Lanka at different scales [7]. However, these methods have limitations that hinder their effectiveness. Larvivorous fish and copepods require suitable aquatic habitats with appropriate water quality and vegetation. Limited availability of suitable habitats may restrict their use in certain areas. Larvivorous fish and copepods may not effectively target all mosquito species or instar stages. Some mosquito species can evade predation or utilize alternative breeding sites, reducing the overall efficacy of biological control [8]. Use of endoparasitic ciliates on the other hand has some limitations such as they are limited to a specific host range and target particular mosquito species or life stages [9]. This limitation restricts their effectiveness as a universal mosquito control solution. Further, endoparasitic ciliates may have complex life cycles involving multiple hosts, making their mass production and implementation challenging and time-consuming [10, 11]. Therefore, new tools in terms of biological means should be evaluated time to times for use in the integrated vector control approaches.

Dengue infection is mainly transmitted by *Aedes* mosquitoes that are considered to be container breeders in micro breeding habitats. However, some recent studies have identified that man-made structures at gardens and recreational areas in the means of ponds, tanks and other ornamental water bodies provide considerable contribution as vector breeding site [12].

Carnivorous plants have fascinated scientists with their insect-capturing ability. Genus *Utricularia* is a carnivorous angiosperm belonging to the family Lentibulariaceae, comprised of approximately 235 species [13]. They are commonly known as bladderworts. The carnivorous genus *Utricularia* harbours many freshwater species which have the potential to trap and utilize a wide range of aquatic invertebrate prey. These plants occur in every continent except Antarctica and some of the arid regions and oceanic islands [14]. They usually grow in nutrient-poor shallow habitats with standing waters like small lakes, ponds, oligotrophic marshes, and their distribution is highly fragmented [14]. A wide range of prey is caught by aquatic *Utricularia* species [15]. Some studies have indicated the predacious efficacy of carnivorous plants against mosquito larvae as a potential control solution [16]. Unlike other biological based control approaches, use of *Utricularia* species has its unique advantages for mosquito larval control mainly *Utricularia* species offer potential benefits as a natural, self-sustaining, and ecologically compatible control measure for various mosquito species and life stages. *Utricularia* species have a global distribution, providing the opportunity for local availability and adaptation to diverse mosquito habitats [14, 17, 18].

However, the effectiveness of using these carnivorous plants in controlling the populations of disease vectors with aquatic developmental phase, such as mosquitoes has not been evaluated in Sri Lanka. Therefore, this study was conducted as the first-ever effort in Sri Lanka to evaluate the carnivorous potential of commonly found *Utricularia* species on medically important *Aedes aegypti* mosquito under laboratory setup.

## Method

### Mosquito colony conditions and larval rearing

*Aedes aegypti* eggs were obtained from the laboratory colony (F10 generation) maintained at the Department of Parasitology, Faculty of Medicine, University of Kelaniya, Sri Lanka, at 27 ± 2 °C and 75 ± 5% humidity, under 12 h: 12 h (light: dark) photoperiod. Experimental eggs were hatched in filtered double distilled water, stimulated by the multiple-immersion clue. The eggs were dipped in a 40–50 °C water bath, which was allowed to cool after boiling, in order to simulate oxygen fluctuations which, facilitate the egg hatching process. After 1 h, early instar larvae were sorted carefully using a pasteur pipette and transferred into larval rearing trays (25 × 25 × 7 cm) containing 500 mL of dechlorinated water. Each container harboured 750 larvae. The larvae were fed in the morning (08.30 h) with a daily dose of standard larval diet optimized previously, comprising of tuna meal, bovine liver powder, and brewer’s yeast [19, 20]. Larval food was added on a per-capita basis at 1.33 × 10^− 2^ mg per larva [21] both to the larval rearing trays and during the experimental trials. Excessive food, fecal matter, and debris in the larval trays were removed every day before adding the morning diet dose using pasteur pipettes, to maintain satisfactory water quality levels for larval development.

### Collection of bladderworts

Fresh samples of *Utricularia* species were collected from the littoral of freshwater ponds (whole plant and segments approximately 25–40 cm in length) in Dankotuwa (N; 7.32950, E; 79.9347), North Western Province (Location 1) and Kandy (N; 7.26216, E; 80.59601), Central Province (Location 2) of Sri Lanka. Collected specimens were transported live to the laboratory at the Department of Parasitology, Faculty of Medicine, University of Kelaniya, Ragama, Sri Lanka. Identification was authenticated by the National Herbarium, Department of National Botanical Gardens, Peradeniya, Sri Lanka. The species collected from location 1 was identified as *U. aurea* Lour. The specimen collected from location 2 was authenticated up to the genus level as *Utricularia* sp. These specimens were deposited at the National Herbarium, Sri Lanka as voucher specimens for future use.

### Cultivation of bladderworts

Collected bladderworts were first dipped in 4 L of water containing 1 ml of 1% methylene blue for 2 h followed by washing with de-chlorinated water twice, to eliminate contamination and possible prey from their surface, such as invertebrate grazers and attached protozoa. The two species of bladderworts were kept separately in glass tanks with muddy sediment (obtained from the collected locations) at room temperature for two weeks, for acclimation to the laboratory conditions prior to the main experiment.

### **Predation of*Aedes aegypti*larvae by two selected carnivorous plants**

Early instar (i & ii) and late instar (iii & iv) larvae of *Ae. aegypti* were selected as prey for evaluating the predatory efficacy of bladderworts. The experiment design was set with 10 larvae (both early and late instars separately) in 250 ml of water with bladderwort containing approximately 100 bladders in a plant segment.

Middle segments of bladderworts from each species (after removing the decaying parts) were taken. The number of bladders in the segments were enumerated using a binocular dissecting microscope. The plant segments containing approximately 100 bladders in each segment were taken for the present experiment. The length of the segments in two species was different since the number of bladders length of the leaf nodes to represent 100 bladders were different in two species used in the experiment. In general, arrangement of bladders in *U. aurea* was very closer compared to the *Utricularia* sp. On average, 5–10 cm segments were used for *U. aurea* while segments ranging 25–40 cm were required for *Utricularia* sp. The average trap size of the largest bladder was 0.8–1.0 mm and 0.4–0.6 mm in *U. aurea* and *Utricularia* sp., respectively. The approximate age of the used leaf nodes from both species were ranged 4–6 weeks.

A total of 50 replicates were conducted (both early and late larval stages, separately) for both bladderworts. Controls were incubated without plants, under the same conditions as described above. The larvae were fed on a per-capita basis once a day in the morning, with 1.33 × 10^− 2^ mg per larvae using the standard diet described above [21]. The above experimental set-up was carried out separately for both species. The survival status of larvae was recorded daily until death or adult emergence. The bladders were examined daily under a dissecting microscope mounted with a microscopic camera (20x), and using hand-held lenses. The larval prey was attributed to direct predation when they found whole or partially inside of the bladders [16].

### Predatory behaviour of carnivorous plants

The predatory behaviour of the carnivorous plants was recorded using a camera (ScopeImage HDCE-X5) mounted to a compound light microscope. For this, 5–10 bladders were focused and fixed within the optical field. The activity in the focused field was recorded for 2 h, and the important events in the records were observed by fast-forwarding the record at 5-minute intervals.

### Statistical analysis

Statistical analyses were conducted in Statistical Package for the Social Sciences (SPSS, version 23). The effects of two field-caught bladderworts on the survival of early instar (i & ii) and late instar (iii & iv) larvae of *Ae. aegypti* were evaluated using the Cox-proportional Hazard model [16]. The Mantel-Cox logrank test was used as the statistical method to compare the survival curves generated for the survival of *Ae. aegypti* larvae in the presence and absence of two field-caught species were evaluated separately. The Hazard Ratio (HR) was calculated along with the 95% confidence interval to evaluate the extent of the predatory efficiency over *Ae. aegypti*.

## Results

### **Predation of*Ae. aegypti*early instar (i & ii) larvae by two bladderworts**

**Fig. 1:**
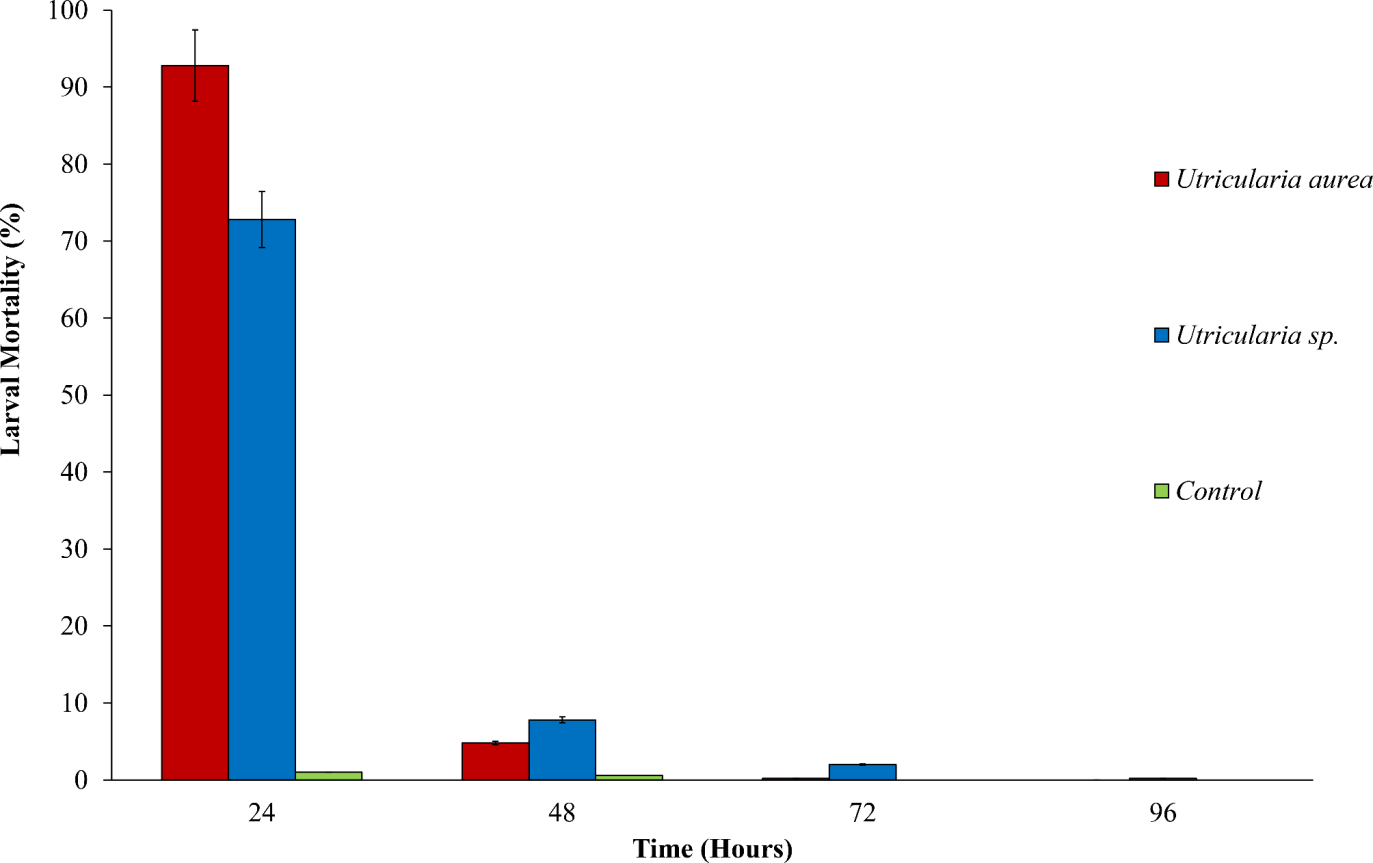
The percentage mortality of *Ae. aegypti* early instar larvae with *U. aurea* and *Utricularia* sp. at different exposure periods

The early instars of *Ae. aegypti* larvae exhibited 92.8% mortality rate when exposed to *U. aurea* within the first 24 h (Fig. 1). Subsequently, 4.8% and 0.2% larval mortalities were observed after 48 and 72 h of exposure, respectively. Overall, 97.8% of larval mortality was observed in *U. aurea* after 72 h after which no further larvae capture events were observed. A negligible mortality rate (≤ 1%) was observed in the controls, which were monitored in parallel to the experiments. A similar trend was observed for *Utricularia* sp. also with early instar larvae of *Ae. aegypti* (Fig. 1). At 24 h of exposure, 72.8% of mortality was observed, followed by additional 7.8% and 2% at 48 and 72 h of exposure, respectively.

The cumulative mortality rate after 72 h in *Utricularia* sp. was 82.6%, which is lower compared to *U. aurea*. Larvae mortality in this treatment was observed until 96 h after initial exposure. The overall cumulative mortality after 72 h however, increased only by 0.2–82.8%. The Cox-regression model was observed to be significant denoting a good fit for both *U. aurea* (Likelihood ratio test: 369.450; *X*^2^ = 13.46; *df* = 1; *P* < 0.001) and *Utricularia* sp. (Likelihood ratio test:317.562; *X*^2^ = 14.25; *df* = 1; *P* < 0.001). According to the Mantel-Cox log-rank test, the presence of both *U. aurea* and *Utricularia* sp. significantly reduced the larvae numbers of *Ae. aegypti* under laboratory conditions compared to the control (Hazard Ratio [HR] = 60.71, CI; 5.69–999.25, *P* = 0.004 and *HR* = 54.42; *CI*;3.04–975.43, *P* = 0.007, respectively). Based on the Hazard Ratio values, *U. aurea* (HR = 60.71) showed a higher predatory potential on early instar larvae of *Ae. aegypti* compared to *Utricularia* sp. (HR = 54.42). The predatory potential of *U. aurea* was significantly different from *Utricularia* sp. (*P* = 0.022).

### **Predation of*Ae. aegypti*late instar (iii & iv) larvae by two bladderworts**

**Fig. 2:**
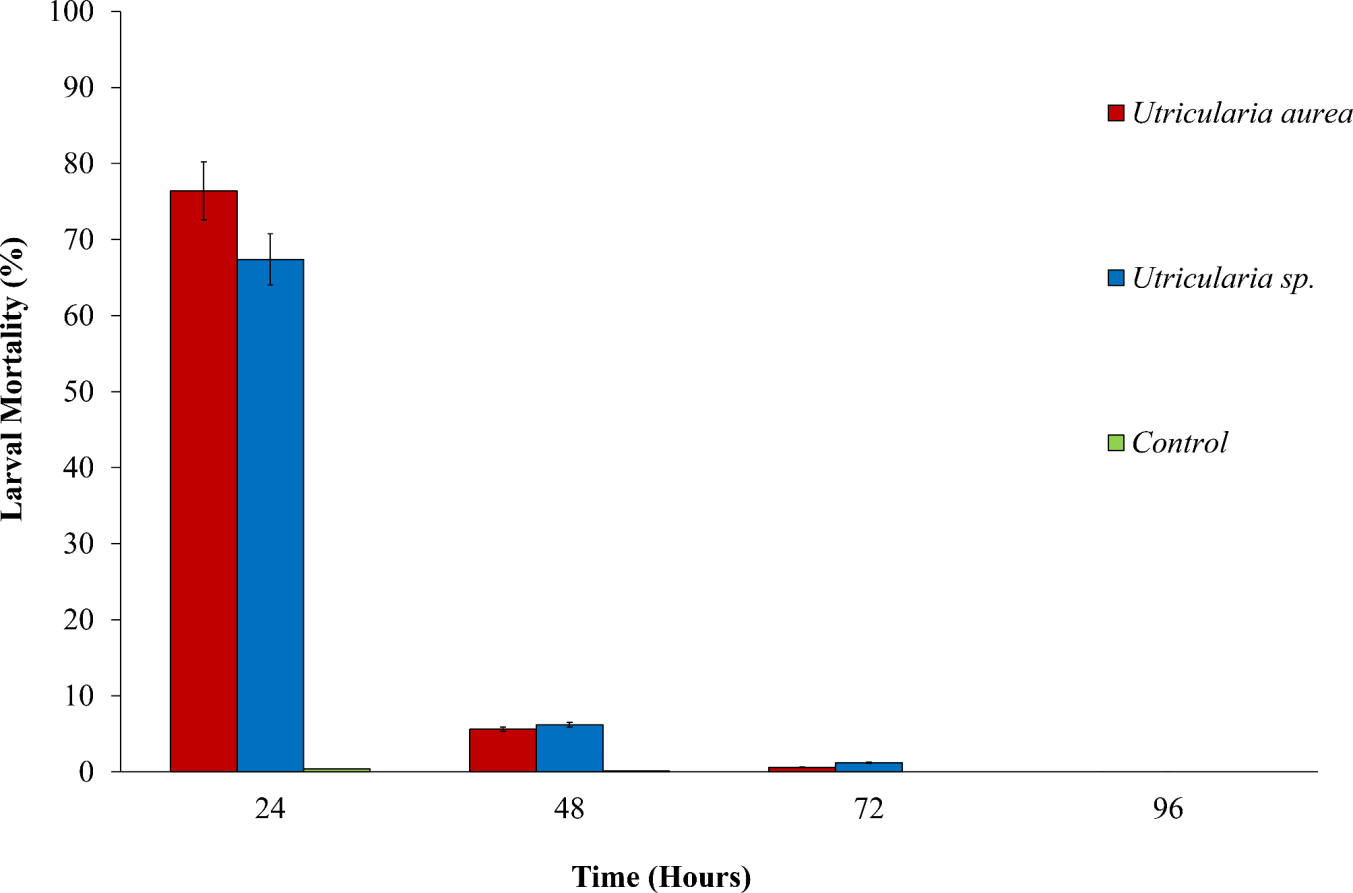
The percentage mortality of late instar *Ae. aegypti* larvae with *U. aurea* and *Utricularia* sp. at different exposure periods

A cumulative mortality rate of 82.6% was observed at the end of 72-hours in *U. aurea* treatments with late instar larvae (Fig. 2). The highest predation of 76.4% was observed at the end of the first 24 h of exposure, followed by 5.6% and 0.6% mortalities detected in the next 48 and 72 h of incubation. In the case of *Utricularia* sp., the first 24 h of incubation exhibited the highest mortality of 67.4%, followed by 6.2% and 1.2%, respectively, at 48 and 72 h of exposure. The cumulative mortality after 72 h thus reached 74.8% (Fig. 2). No further predation was observed after 72 h. Based on the Mantel-Cox log-rank test and the cox-regression model, late instar *Ae. aegypti* larvae exposed to *U. aurea* showed significantly higher mortality (*HR* = 45.02; *CI*: 5.96–850.51, *P* = 0.017; Likelihood ratio test: 280.620; *X*^*2*^ = 22.71; *df* = 1; *P* < 0.001).

The late stages of *Ae. aegypti* larvae exposed to *Utricularia* sp. also indicated significant mortality rates (*HR* = 228 36.69; *CI*: 6.75–704.51, *P* = 0.022) based on the Cox-regression model (Likelihood ratio test: 242.158; *X*^*2*^ = 22.92; *df* = 1; *P* < 0.001). As shown by the Hazard Ratio values, *U. aurea* exhibited significantly higher predatory potential (*P* = 0.03) on late instar *Ae. aegypti* larvae (HR = 45.02), compared to *Utricularia* sp. (HR = 36.69). An edited short video indicating the predatory behaviour of *U. aurea* is included in the Additional field 1; Video 1.

## Discussion

Aquatic *Utricularia* are widely distributed globally [17], and occur almost throughout the *Ae. aegypti* distribution range. Specific examples include *U. macrorhiza* distributed in North America, and North-Eastern Asia [17], as well as *U. reflexa* in Uganda [18]. The cosmopolitan distribution singles out *Utricularia* species as a viable option for the biological control of aquatic invertebrates such as medically important mosquitoes [16, 22, 23]. Several *Utricularia* predators may thrive outside of their natural habitat [18, 22–26] and thus may be applied to the control of container-breeding species [16]. Dengue vector mosquitoes mainly breed in small man-made containers or environments that are with limited natural preys [16]. However, the application of *Utricularia* species as a biological control agent for mosquito larval control has been relatively understudied.

The predation potential of bladderworts varied with the size of larvae (larval stage) and the period of exposure to the carnivorous plant. The larval mortality is caused by suffocation due to anoxic conditions inside the bladders. The two species used in the present investigation preyed on several larval instars of *Ae. aegypti*. There is a possibility for the bladderwort in getting preyed upon fourth instars and pupae [27], but the bladder size observed was much smaller than those mosquito developmental stages. Therefore, capturing of larvae by the traps depends on the bladder size and development stage of the mosquito larvae. The size of the biggest traps in the bladders in both species namely; *U. aurea* and *Utricilaria* sp. were ranged from 0.8 to 1.0 mm and 0.4–0.6 mm in *U. aurea* and *Utricularia* sp., respectively. The majority of first and second-instar larvae had fully trapped inside the bladders and third instars had trapped either completely or partially. Some of the late stages (iii and iv instars) of larvae had trapped at their siphons or anal segments. Therefore, the carnivorous potential of *Utricularia* species can be expected up to the fourth-instar larvae of *Ae. aegypti.*

In the present study, the trapping rate of bladderworts was higher within the first 24 h and mortality rate of larvae were reduced thereafter. Similar results have been indicated by *U. minor* and *U. macrorhiza* against *Ae. aegypti* and *Ae. albopictus*, respectively by eliminating larvae within 4–6 days [16, 28]. Overall, decrease in prey number could be linked with decrease in trapping rate and possible trapping of very small traps with relatively big mosquito larvae.

In this experiment, the mortality of larvae in the experimental setup was monitored until 72 h. Initially 10 *An. stephensi* larvae were introduced in to each experimental trial and no larvae were introduced after 24 and 48 h of observation. Therefore, larval mortality was accounted as the percentage of dead larvae out of the survived individuals at each observational period. Similar procedure had been used by previously published studies relevant to the carnivorous ability of *Utricularia* species against mosquito larvae [16]. However, larval mortality could have been more if dead larvae were replaced with live once after each observational period. Therefore, this we identify as a limitation in this study. One possible reason for the reduced efficacy of trapping rate in bladderworts after the first insect trapping is related to the trap resetting process. Bladderwort traps are vacuum-driven structures that rely on rapid changes in internal pressure to capture prey. After capturing an insect, the trap needs to reset and reopen to be able to capture more prey. This resetting process takes time and energy for the plant. It has been suggested that the energy expenditure and the physical wear and tear associated with trap resetting might result in reduced efficacy in subsequent trapping events [29]. It was observed that bladderworts could capture a certain number of prey items before their trapping efficiency decreased. The authors speculated that the reduction in efficacy might be due to the depletion of digestive enzymes or the accumulation of indigestible material within the traps. These factors could impair the trap’s ability to properly digest and assimilate nutrients from subsequent prey items.

Unlike other aquatic predators, bladderworts do not elicit prey preference based on the composition of prey material [30]. The mechanism of trapping is raised in the bladder door when the external trigger hairs are stimulated [31]. Besides, trapping insects by *Utricularia* is quickest compared to predation by animals [32]. The traps have the potential to capture and preyed on multiple animals one after the other and multiple prey animals can be captured within a single suction swirl [33]. In *Utricularia*, no morphological change or growth is required before a second capture [34]. These properties advocate the larvivorous potential of genus *Utricularia* which would be used as a biological control agent for medically important mosquito larvae.

A laboratory based study conducted with *U*. *macrorhiza* has indicated the preying of *Ae. aegypti* larvae from first to the third instar [16]. A study conducted using *U. australis* has indicated that this bladderwort could be used as a biocontrol agent against *Ae. albopictus* larvae due to its ecological plasticity, broad distribution, ability to thrive in small containers and good overlap with the habitat preference of both dengue vectors, *Ae. aegypti* and *Ae. albopictus* [26]. Further, *U. minor* has also indicated a potential to eliminate *Ae*. *aegypti* larvae within six days of exposure in artificial containers [28] indicating the suitability of these carnivorous plant for different breeding site categories ranging from natural to man-made. The present study also documents the larvivorous potential of both *U. aurea* and *Utricularia* sp. against larval stages of *Ae. aegypti* from first to fourth instars.

The predation potential of the two tested in the present study was not similar. In general, *U*. *aurea* indicated a higher predation efficiency of the two tested *Utricularia* species. Bladder size may be a significant determinant for larvivorous potential [32] and our original observation also confirmed that the bladder size of *U. aurea* was larger than *Utricularia* sp. evaluated in this study. In addition, resetting time after the first trap is required and it could be varied with the species [35]. In general, such as *U*. *vulgaris* has a resetting time of 15–30 min [36]. Further, other factors such as temperature [37], trap age, and the separation length of the plant part from its natural site would also impact on the larvivorous potential and efficacy [38]. Therefore, the difference in the larvivorous potential of these two may be due to one or more factors as described above. Hence, important factors that determine the larvivorous potential of bladderworts should be investigated to find out the best and stage of the plant to be introduced into a breeding habitat of interest.

Larval density is another important factor as it determines the constant contact between the predator and prey. The appropriate number of predators to be used or predator to larvae ratio is important to know, as it governs the capacity of the control method [39]. Previous studies have shown that the bladder to larval ratio and small water volumes are not limiting factors in the application of *Utricularia* species. [16]. However, the majority of these studies have used 10:1 bladder to larval ratio that resulted higher larvivorous potential. Therefore, the present experimental setup also maintained 10:1 ratio of bladder to larval counts.

In the present study, only two were collected from two different provinces in Sri Lanka and authentication of one species was not possible to reach to the species level. Therefore, island-wide surveys to determine the presence of *Utricularia* species and their potential for mosquito control would provide better candidates to be used for vector control interventions at different breeding habitat types. However, *U. aurea* which indicated the highest larvivorous efficacy in the present study is an indigenous aquatic plant to Sri Lanka that is seen mostly in dry regions with low altitudes. It is a perennial aquatic floating herb without roots that commonly grow in tanks, ditches, stagnant water, pools and swamps [40]. Therefore, this could be an ideal candidate to be used for natural breeding sites for dengue mosquitoes and structures with water (indoor/outdoor) built for recreational/aesthetic purposes.

There are prerequisites in the use of an organism in biological control. Easy in maintaining the stock, overlapping with the prey distribution, surviving in prey habitats, auto-reproduction in sustaining, and cost-effectiveness are main concerns [41]. There are some evidences that bladderworts attract mosquitoes [42] and induce oviposition of adult female mosquitoes. [43]. Therefore, carnivorous potential of bladderworts could be an ideal biological candidate for mosquito larval control. Further, aquatic vegetation may attract dragonflies for oviposition which enhances further biological control by nymph stages of dragonflies [44]. Since these are flowering plants, they ultimately enrich the recreational value and contributes to ecological services such as the attraction of various pollinators like bees and butterflies. On assumption, 10 segments of *U. aurea* of 5–10 cm in length would be sufficient to eliminate > 97% of early stage and 82% of late instar *Ae. aegypti* larvae in a breeding site containing 2.5 L of water. However, it is recommended to evaluate the field application of this approach and the feasibility of this approach to be used in the integrated vector management programmes.

## Conclusion

*Utricularia aurea* is a competent biological predator against early (I & ii) and late instar (iii & iv) larvae of *Ae. aegypti*. Early instar stages were highly susceptible to predation compared to later instars. However, > 70% of cumulative predation after 72 h of exposure was observed for early and late instars in both *Utricularia* species tested in the present study. Therefore, in a natural system, it could be assumed that the earlier instars may be trapped initially and 70% of the non-trapped ones will be trapped even during the late instar levels. Hence, the adult emergence from the breeding site could be minimal. It is recommended to conduct field-based studies to explore the feasibility and applicability of bladderworts as a control intervention for mosquitoes.

### Electronic supplementary material

Below is the link to the electronic supplementary material.

Additional file 1: video S1: Short video capturing the predation of third instar larvae of *Aedes aegypti* by *Utricularis aurea*

## Data Availability

The datasets of the study are available from the corresponding author upon reasonable request.

## Abbreviations

DO: Dissolved Oxygen
EC: Electrical conductivity
HR: Hazard Ratio
